# Predictive value of blood urea nitrogen in heart failure: a systematic review and meta-analysis

**DOI:** 10.3389/fcvm.2023.1189884

**Published:** 2023-07-31

**Authors:** Siyu Duan, Yuqi Li, Ping Yang

**Affiliations:** ^1^ Second Clinical Medical School, Medical University of Kunming, Kunming, China; ^2^ School of Basic Medicine, Medical University of Kunming, Kunming, China

**Keywords:** blood urea nitrogen, heart failure, all-cause mortality, prognosis, independent predictor

## Abstract

**Background:**

The mortality rate of patients with heart failure (HF) remains high, and when heart failure occurs, blood urea nitrogen (BUN) is involved in the perfusion of renal blood flow. Some studies have shown an association between heart failure prognosis and blood urea nitrogen, but the results of some other studies were inconsistent. Therefore, we conducted a comprehensive meta-analysis to investigate the value of BUN on the prognosis of patients with heart failure.

**Methods:**

A computerized systematic search of all English literature was performed in four databases, PubMed, Cochrane, Embase and Web of Science, from their inception to May 2022. The data of BUN were classified into continuous and categorical variables after passing the inclusion and exclusion criteria. The BUN data of both types were extracted separately into stata15.0 for statistical analysis.

**Results:**

A total of 19 cohort studies involving 56,003 patients were included. When BUN was used as a categorical variable, the risk of death in heart failure was 2.29 times higher for high levels of BUN than for low levels of BUN (*RR* = 2.29, 95% CI:1.42–3.70, *P* < 0.001). The results showed statistical significance in multifactorial and univariate groups, the prospective cohort, and European and Asian groups. When BUN was used as a continuous variable, the risk of death in heart failure was 1.02 times higher for each unit increase in BUN (*RR* = 1.02, 95% CI:1.01–1.03, *p* < 0.001). Subgroup analysis showed statistical significance in retrospective cohort, American and Asian.

**Conclusion:**

High BUN is an independent predictor of all-cause mortality in heart failure. Lower BUN was associated with better prognosis in patients with heart failure.

## 1. Introduction

Heart failure is a critical condition of the cardiovascular system, and in the United States, heart failure is one of the leading risk factors for hospitalization in the elderly, with 1 million hospitalized patients with heart failure according to the statistics of the sample of U.S. inpatients in 2014 (1). Despite increasingly sophisticated treatments for heart failure, nearly half of the patients die within five years after discharge from the hospital (2). Therefore, indicators that monitor the risk of death in heart failure patients since admission are important in determining the prognosis of heart failure. Studies have shown that heart failure is often combined with reduced renal function, and that indicators of renal function can independently predict the risk of death from heart failure (3).

At present, a variety of renal function markers associated with heart failure have been found. Among them, serum creatinine is the most commonly used indicator, which can be filtered through the glomerulus. When creatinine concentration is elevated, renal function deteriorates with an decreased glomerular filtration rate. However, the increase in serum creatinine may also be caused by hypermyolysis. Therefore, serum creatinine cannot fully reflect heart failure (4). Cystatin C is a small protein synthesized by the body that freely filters through the glomeruli to form urinary cystatin C. Urocystatin C is considered to be a sensitive marker of kidney injury in patients with chronic heart failure. However, some studies have shown that elevated cystatin C is not associated with the prognosis of heart failure, which is quite controversial (5).

Blood urea nitrogen is involved in changes in renal perfusion and better reflects the progression of heart failure than traditional indicators such as blood creatinine (6). For every 10 mg/dl increase in blood urea nitrogen, the mortality rate of heart failure patients increases by 21% (7). In a study of patients hospitalized with acute decompensated heart failure (PROTECT), blood urea nitrogen was the strongest predictor of 180-day mortality in patients with heart failure. In the results of the prospective trial of intravenous milrinone for the treatment of chronic heart failure exacerbations (OPTIME-CHF), blood urea nitrogen also predicted the risk of death in patients with heart failure (8, 9). Thus, blood urea nitrogen may be an important independent predictor of short and long-term mortality in patients with heart failure. However, there were some studies with inconsistent results. In the study of Sachdeva et al. (10), BUN had no predictive value for the prognosis of heart failure. In the study of Takaya et al. (11), BUN showed predictive value only for the prognostic of heart failure combined with renal failure. (The predictive value of BUN for heart failure in these studies remained controversial.) Therefore, we found it important to conduct a thorough and detailed meta-analysis to assess the value and role of blood urea nitrogen in predicting the prognostic risk of death in patients with heart failure.

## 2. Methods

### 2.1. Search strategy

We systematically searched all the results from the databases of Embase, PubMed, Cochrane, and Web of Science since their inception until May 2022 by combining subject terms with free words. The search terms were “heart failure” or “heart decompensation” or “cardiac failure” or “myocardial failure” combined with “BUN” or “blood urea nitrogen” or “serum urea nitrogen” (See the appendix for the search strategy).

### 2.2. Inclusion and exclusion criteria

For this study, our inclusion criteria were (1) study type: cohort study; (2) subjects: all patients ≥18 years old and diagnosed with heart failure according to clinical criteria; (3) exposure: blood urea nitrogen levels provided in admitted heart failure patients; (4) outcome measure: all-cause mortality.

Exclusion criteria included (1) animal studies and *in vitro* studies; (2) systematic reviews, conference abstracts, letters, and case reports; (3) non-English literature; and (4) duplicate studies (articles with multiple occurrences or identical data).

The retrieved studies were imported into EndNote for literature screening. After removing duplicate studies, all literature was screened based on title, abstract, and data type. The literature was screened separately by two researchers and categorized as eligible, uncertain, or ineligible based on the inclusion and exclusion criteria. The full texts of articles that were jointly classified as “eligible” by both researchers was summarized for our review and assessment. Therefore, a third researcher was not required to verify the inconsistency of the literature.

### 2.3. Data extraction and literature quality evaluation

After the search was completed, we used an Excel spreadsheet to summarize the basic information in the 19 articles and extracted the first author, year of publication, study design, study type, study location, follow-up time, all-cause mortality, and univariate and multifactorial analyses of blood urea nitrogen levels. In terms of literature quality assessment, we fully considered the impact of the following six aspects: (1) study participants, (2) loss to follow-up, (3) measurement of risk factors, (4) measurement and control of confounding variables, (5) outcomes measurement, and (6) analyses and reports. Each aspect was classified to a high, moderate, or low risk of bias. In this paper, a study was considered to be of high quality if four or above aspects including the loss to follow-up and the confounders were evaluated with low risk of bias (14).

### 2.4. Statistical analysis and graphing

RR(relative risk) and 95% CI(credibility interval)were used to evaluate the effect of blood urea nitrogen on the prognosis of heart failure based on the data with key characteristics from the data table, and the evaluation index was the *RR* values pooled across studies. The heterogeneity of included studies was investigated by the Cochran *Q*-test. Fixed-effects and random-effects models were selected by *p*-value and *I*^2^ value in the forest plot. If *I*^2 ^> 50% and *p* < 0.1, the random-effects model was adopted; if *I*^2^ < 50% and *p* > 0.1, the fixed-effects model was employed (15). When heterogeneity was large, subgroup analysis was conducted by study type, single and multiple factors, and different continents to explore the sources of heterogeneity. We performed a sensitivity analysis to determine whether the exclusion of any of the articles would affect the stability of the results. The publication bias of the literature was analyzed by generating Begg's and Egger's tests. The Begg's test is based on the rank sum test, and the Egger's test is based on the regression test. Usually, the *p*-value can be used to determine whether there is publication bias. If the *P* > 0. 05, there is no publication bias. If *p* < 0.05, the trim-and-fill method was required to correct publication bias. The trim-and-fill method was used to trim the asymmetric part of the funnel plot and fill in the trimmed part along the center of the funnel plot. Finally, the new effect size was pooled to check whether it affected the result robustness. All statistical analyses were completed using Stata 15.0 software with a test level of α = 0.05.

## 3. Results

### 3.1. Literature search results

**Figure 1:**
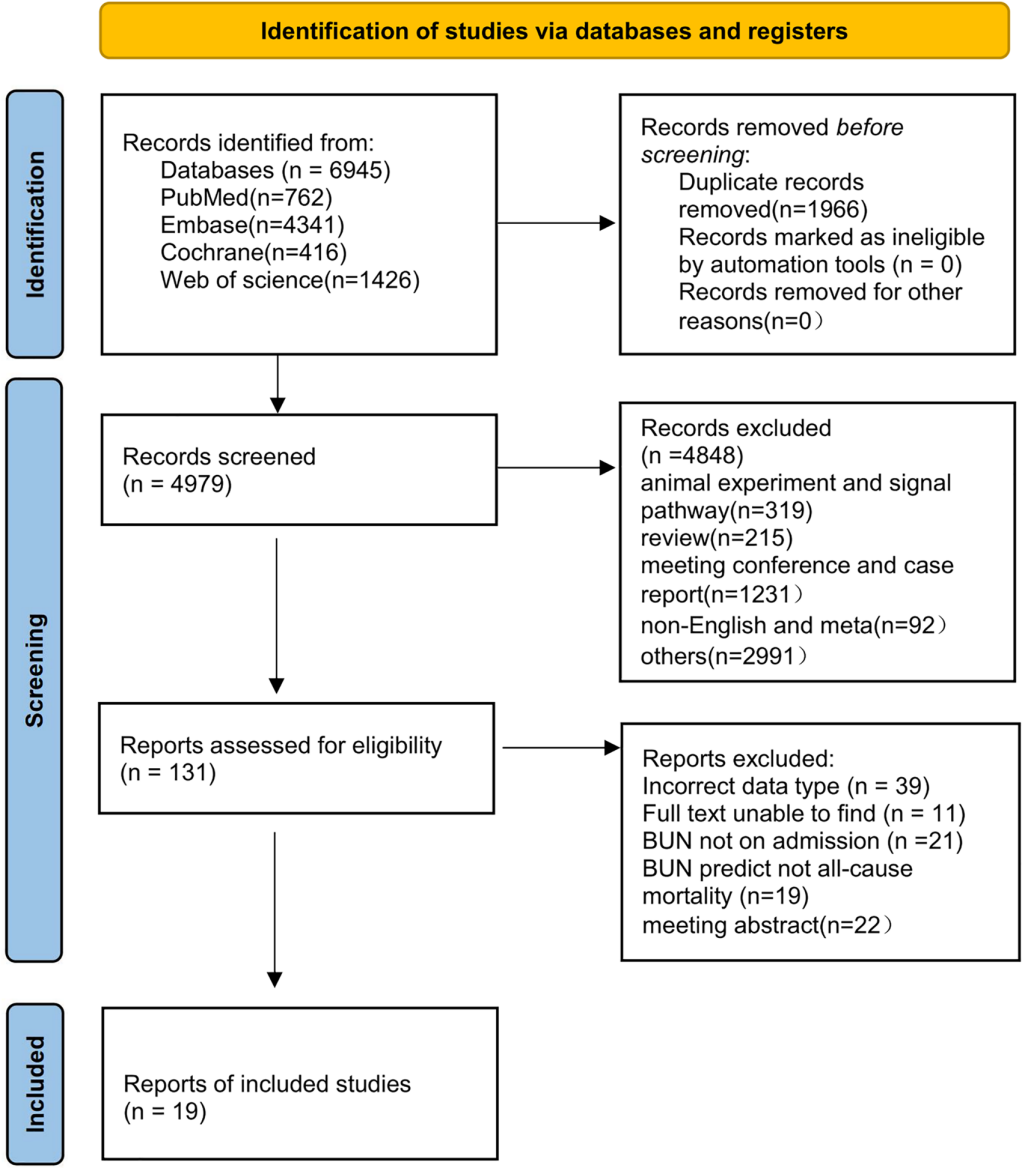
Literature screening process.

The initial search yielded 6,945 papers, and 1,966 duplicates were excluded through the initial screening process in Figure 1. Among the remaining 4,979 papers, a series of publications unrelated to heart failure research, such as conference studies and case reports, were excluded, and 131 articles were obtained. A re-screening of data types and outcome indicators of blood urine nitrogen was performed on the 131 articles, and 19 (16–33) of them were finally included (Figure 1).

The basic information and literature quality evaluation of the included studies is shown in Table 1. The 19 papers published from 2004 to 2022 included patients with heart failure from different countries, most of whom were male and ranged in age from 55 to 78 years. We divided the blood urea nitrogen data into two groups, 10 for continuous variables and 10 for categorical variables. Of these, two papers included data from two cohort studies and one article contained both continuous and categorical variables. The QUIPS (Quality In Prognosis Studies) evaluation results are shown in Table 2. Through quality risk evaluation using the QUIPS tool, nine articles were rated as low risk in terms of the confounders and the loss to follow-up, and as medium risk in terms of one or two of the remaining four aspects. These nine articles were considered to be of high quality. The remaining 11 articles had a moderate risk in terms of the loss to follow-up or the confounders and in terms of at least three of the remaining four aspects. These 11 articles were considered to be of medium quality.

**Table 1:** Basic information of included literature.

Author	Study Type	Country	Recruitment Time	Follow-Up (Years)/(median or mean)	Sample Size	Female (%)	Cohort Characteristics	NT-proBNP(mean(SD)/mean(IQR)	NYHA (n/mean(SD)	Age (Years)(Mean ± SD)/median (IQR)	LVEF (%)(Mean ± SD)/median (IQR)	Outcome	QUIPS
Aronson et al. (16)	pc	USA	6 months	0.95	541	30	ADHF	/	I or II 24	63 ± 14	/	All-cause mortality	Moderate
									III 170				
									IV 111				
Cauthen et al. (17)	rc	USA	1996.5–2005.12	1.21	444	70	AHF	/	/	59 ± 11	38 ± 10	All-cause mortality	Moderate
Sachdeva et al. (10)	pc	USA	1999–2009	2.00	1,215	75	AHF	575 (190–1,300)	III 552	53 ± 13	23 ± 7	All-cause mortality	Moderate
									IV 388				
Scrutinio et al. (18)	pc	Italy	2001.1–2008.4	3.47	802	21	AHF	3,978 (5,395)	2.5 (0.6)	64 ± 12	/	All-cause mortality	Moderate
Miura et al. (19)	rc	Japan	2009.11–2011.2	5.00	337	48.7	AHF	/	III and IV325	75.99 ± 11.93	45.2 ± 16.5	All-cause mortality	High
Ren et al. (12)	rc	China	2012.1–2016.1	2.60	652	59.6	ADHF	752 (291, 1,576)	III 141	73.9 ± 7.8	50.8 ± 12.8	All-cause mortality	Moderate
									IV 277				
Khoury et al. (21)	rc	Israel	2018.11–2018.1	3.00	4,768	46.2	ADHF	/	/	74. ± 12.7	/	All-cause mortality	Moderate
García-Gutiérrez et al. (22)	pc	Spanish	2013.4–2014.12	0.10	762	49.8	HFpEF	3.37 (1.75–6.03)	I 133	76.91 ± 11.42	27.69	All-cause mortality	High
									I I 262				
									III 248				
									IV 155				
					916	52.2	HFrEF	3.57 (1.98–7.25)	I 126	78.98 ± 9.97	25.32	All-cause mortality	
									II 195				
									III 237				
									IV 141				
Shiraishi (23)	pc	Japan	2013–2015	/	4,351	40.1	AHF	3,867 (1,917–8,741	II 774	74.6 ± 13.1	43.5 ± 15.9	All-cause mortality	High
									III 1,644				
									IV 1,914				
					1,682	44.6	AHF	6,820 (2,908–13,840)	II 617	77.5 ± 12.4	46.3 ± 16.0	All-cause mortality	
									III 1,631				
									IV 2,101				
Wei et al. (24)	rc	China	2019–2021	0.07	1,371	43.3	AHF	/	/	Training /	/	All-cause mortality	
					685	42.2	AHF	/	/	Internal /	/	All-cause mortality	High
					124	45.1	AHF	/	/	External /	/	All-cause mortality	
Rohde et al. (25)	pc	Brazil	2000.8–2004.1	/	779	50	AHF	/	3.5 (0.6)	67 ± 14	42 ± 16	All-cause mortality	Moderate
Ather et al. (26)	rc	USA	2003–2013	10.00	358	99	AHF	1,267 (773–1,475)	/	68 ± 11	/	All-cause mortality	High
Demissei et al. (27)	rc	USA	2010–2014	0.49	2,033	67.1	AHF	3,000 (3,000–3,799)	IorII 344	70.2 ± 11.6	/	All-cause mortality	High
									III 982				
									IV 599				
Nagai et al. (28)	pc	Japan	2012.11–2015.3	2.00	535	50	HFpEF	414 (225–681)	I 4	80 (73–84)	60 (54–65)	All-cause mortality	Moderate
									II 110				
									III 212				
									IV187				
Gioli-Pereira et al. (29)	pc	Brazil	2012–2019	1.00	695	67.6	CHF	149 (54–355)	I 130	55.4 + 12.2	32 + 8.6	All-cause mortality	Moderate
									II 433				
									III 127				
									IV 5				
Parrinello et al. (30)	pc	Italy	2015.1–2015.11	3.00	73	66	HFpEF	/	III 21	76 ± 8.4	58.7 ± 6.7	All-cause mortality	
									IV 52				
					62	43.7	HFrEF	/	III 28	73 ± 11	34 ± 8.2	All-cause mortality	High
									IV 34				
Sujino et al. (31)	pc	Japan	2006.4–2016.6	2.11	2,090	61.9	ADHF	/	/	76.0 (65.0–83.0)	44.0 (31.0–58.0)	All-cause mortality	Moderate
Li et al. (32)	pc	China	2011.1.–2012.9	5.00	3,335	46.8	AHF	/	/	71 (58–79)	62.5 (44)	All-cause mortality	High
Han et al. (33)	rc	USA	2014–2015	/	15,983	45.4	CHF	/	/	74 (64–83)	/	All-cause mortality	Moderate
					14,428	47.6	CHF	/	/		/	All-cause mortality	Moderate

rc, retrospective cohort; pc, prospective cohort; AHF, acute heart failure; CHF, chronic heart failure; HF, heart failure; HFrEF, heart failure with reduced ejection fraction; HFpEF, heart failure with preserved ejection fraction; ADHF, acute decompensated heart failure.

ADHF is a worsening syndrome of AHF.

**Table 2:** Risk of quality scores.

Author	Study participation	Study attrition	Risk factor measurement	Outcome measurement	Studying cofounding	Statistical analysis and reporting	Final quality rating
Aronson et al. (16)	Low	Moderate	Low	Moderate	Moderate	Moderate	Moderate
Cauthen et al. (17)	Low	Moderate	Moderate	Low	Low	Moderate	Moderate
Sachdeva et al. (10)	Low	Low	Moderate	Low	Moderate	Moderate	Moderate
Scrutinio et al. (18)	Low	Moderate	Moderate	Low	Low	Moderate	Moderate
Miura et al. (19)	Moderate	Low	Low	Low	Low	Moderate	High
Ren et al. (12)	Low	Low	Moderate	Low	Low	Moderate	High
Khoury et al. (21)	Moderate	Low	Moderate	Low	Moderate	Low	Moderate
García-Gutiérrez et al. (22)	Low	Low	Moderate	Low	Low	Moderate	High
Shiraishi (23)	Low	Low	Low	Moderate	Low	Low	High
Wei et al. (24)	Low	Low	Moderate	Low	Low	Moderate	High
Rohde et al. (25)	Low	Moderate	Low	Moderate	Moderate	Moderate	Moderate
Ather et al. (26)	Low	Low	Low	Low	Low	Moderate	High
Demissei et al. (27)	Low	Low	Moderate	Low	Low	Low	High
Nagai et al. (28)	Low	Low	Moderate	Moderate	Moderate	Moderate	Moderate
Gioli-Pereira et al. (29)	Moderate	Moderate	Low	Moderate	Low	Low	Moderate
Parrinello et al. (30)	Low	Low	Moderate	Low	Low	Moderate	High
Sujino et al. (31)	Moderate	Moderate	Low	Low	Low	Moderate	Moderate
Li et al. (32)	Low	Low	Low	Low	Moderate	Moderate	High
Han et al. (33)	Moderate	Low	Moderate	Moderate	Moderate	Low	Moderate

High quality: risk of bias was rated low on at least four of the six domains and was rated low for both study attrition and study confounding.

Moderate quality: risk of bias was rated low or moderate on at least four of the six domains and was rated moderate for both study attrition or study confounding.

### 3.2. Effect of blood urea nitrogen as a categorical variable on the prognosis of patients with heart failure

**Figure 2:**
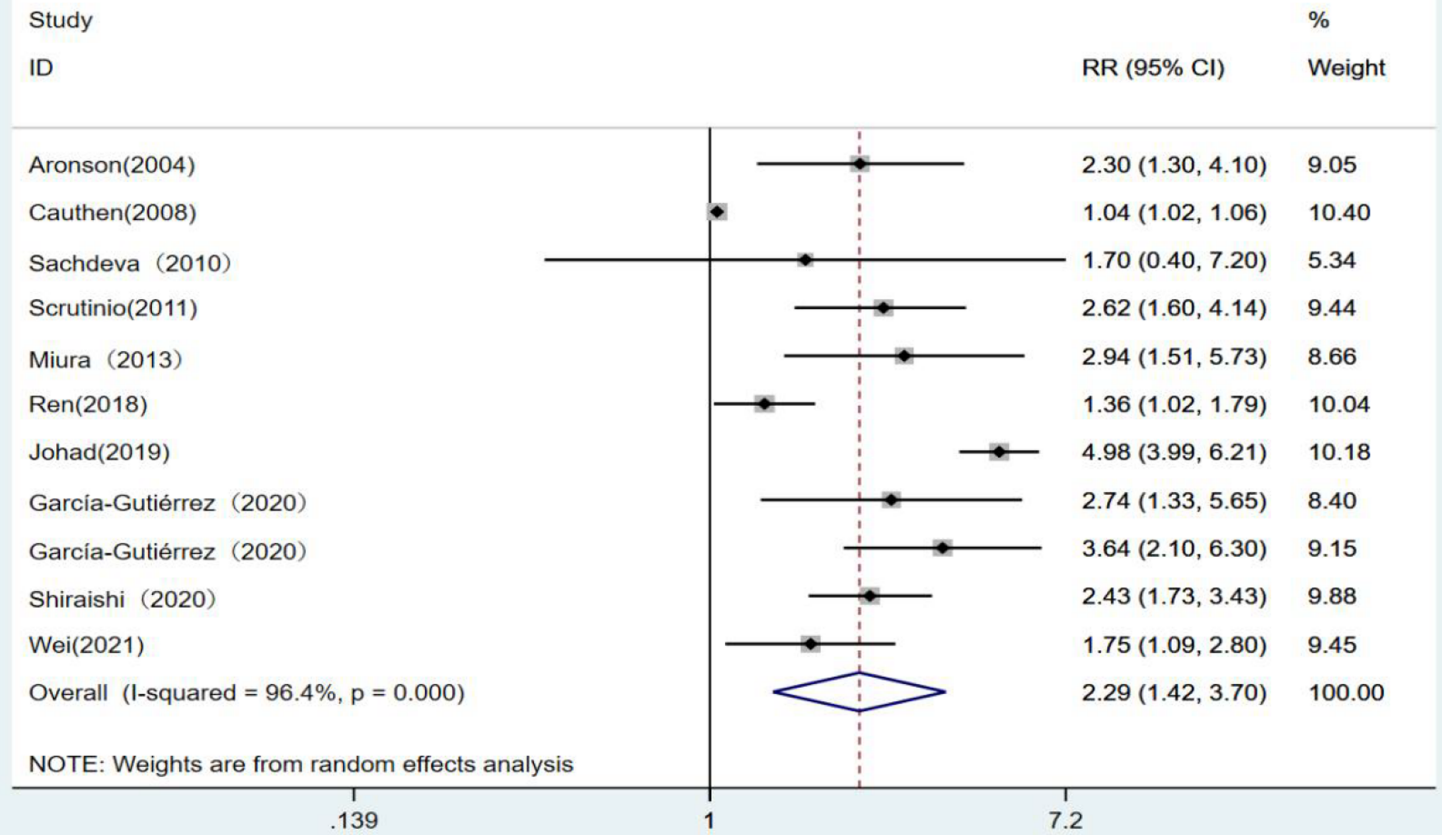
Forest plot of blood urea nitrogen levels on prognosis of heart failure using the random-effects model; heterogeneity test using *Q*-test and *I*^2^ statistic. CI, confidence interval; RR, relative risk.

With 10 articles on the categorical variable, the pooled results revealed that the risk of death from heart failure was 2.29 times higher in the high-level group than in the low-level group for blood urea nitrogen (*RR* = 2.29, 95% CI: 1.42–3.70, *P* < 0.001), and the difference was statistically significant. Since *I*^2^ = 96.4%, a random-effects model was chosen for analysis (Figure 2).

#### 3.2.1. Subgroup analysis

To explore the sources of heterogeneity in outcomes, we conducted subgroup analyses on 10 studies by three subgroups including single and multiple factors, different cohort study types, and continents.

**Figure 3:**
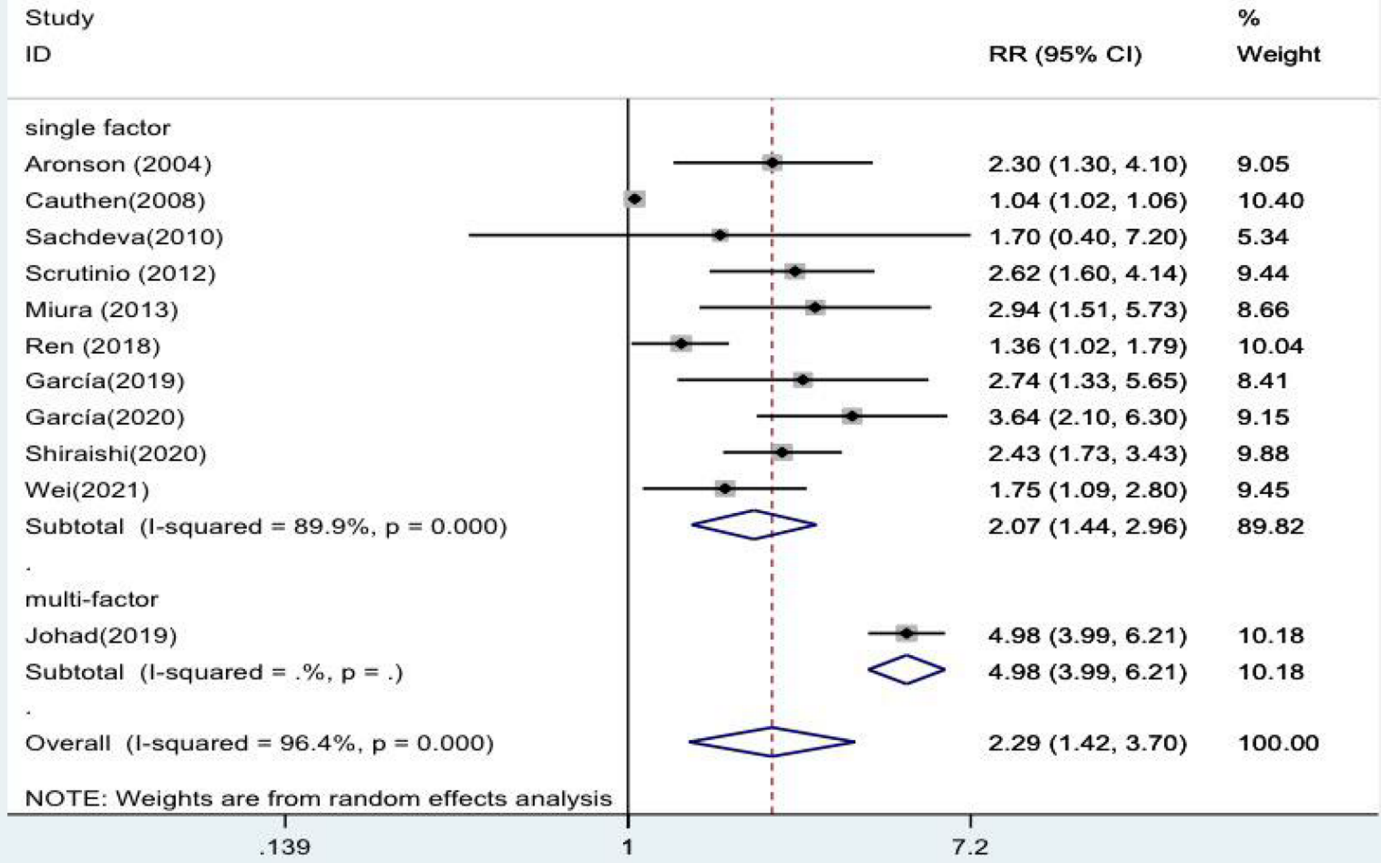
Forest plot of the comparative analysis of blood urea nitrogen levels on the prognosis of patients with heart failure using the random-effects model (single factor vs. multiple factor); heterogeneity test using *Q*-test and *I*^2^ statistic. CI, confidence interval; RR, relative risk.

The forest plots ultimately showed statistically significant results when stratified analyses were performed for single and multiple factors. The risk of death from heart failure with high levels of blood urea nitrogen was 2.07 times higher in the single-factor group than in the low-level group (*RR* = 2.07, 95% CI:1.44–2.96, *p* < 0.001). The risk of developing heart failure with high levels of blood urea nitrogen was 4.98 times higher in the multiivariate group (*RR* = 4.98, 95% CI:3.99–6.21, *p* < 0.001 (Figure 3).

**Figure 4:**
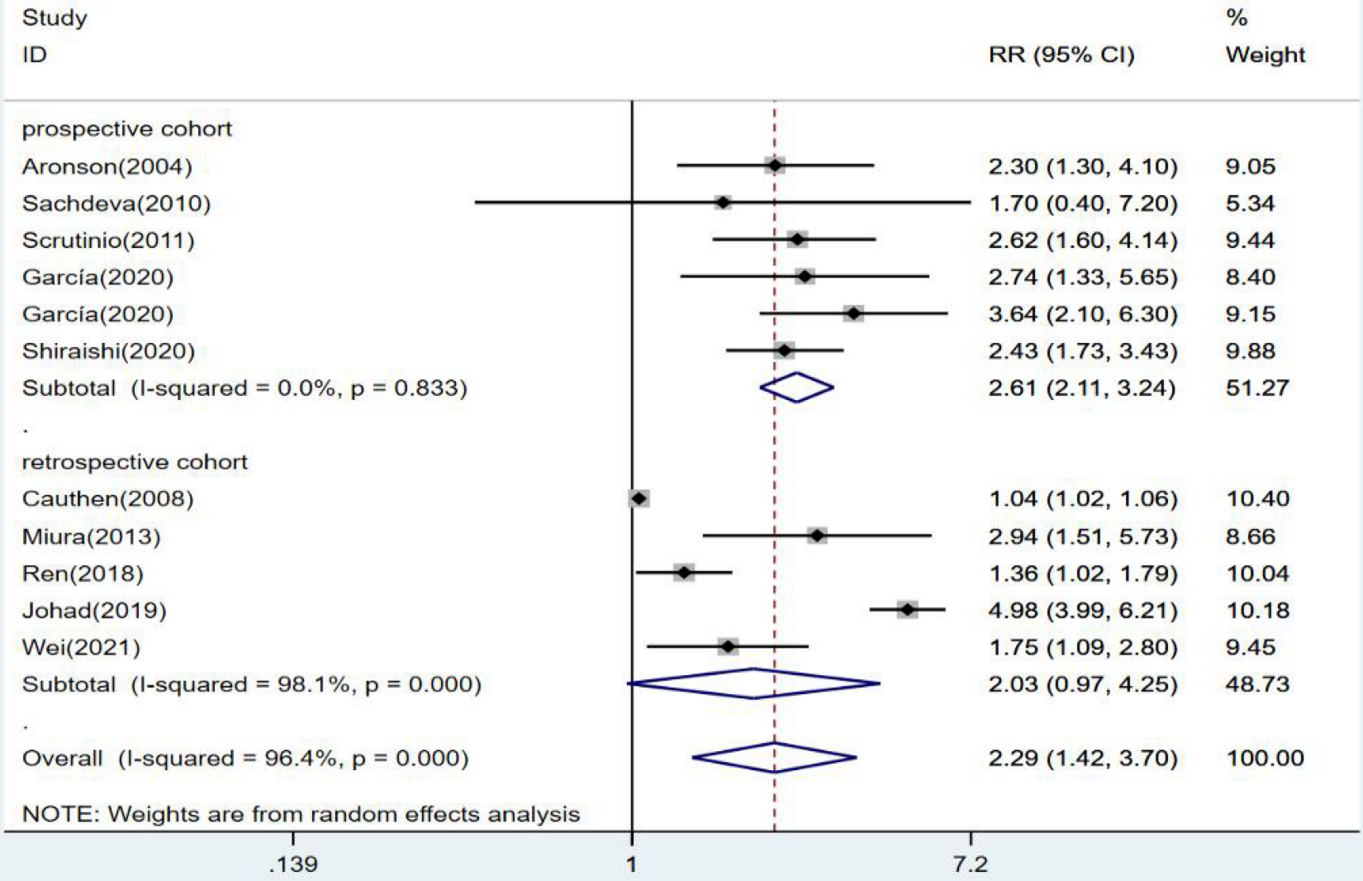
Forest plot of the comparative analysis of blood urea nitrogen levels on the prognosis of patients with heart failure using the random-effects model (prospective vs. retrospective); heterogeneity test using *Q*-test and *I*^2^ statistic. CI, confidence interval; RR, relative risk.

The results were statistically significant in prospective cohort studies when subgroups were defined by study type. The risk of death from heart failure was 2.61 times higher in the group with high levels of blood urea nitrogen than in the group with low levels (*RR* = 2.61, 95% CI:2.11–3.24, *p* < 0.001). In contrast, in retrospective cohort studies, high and low levels of blood urea nitrogen had no statistical significance in predicting death from heart failure (*RR* = 2.03 95% CI:0.97–4.25, *p* = 0.06) (Figure 4).

**Figure 5:**
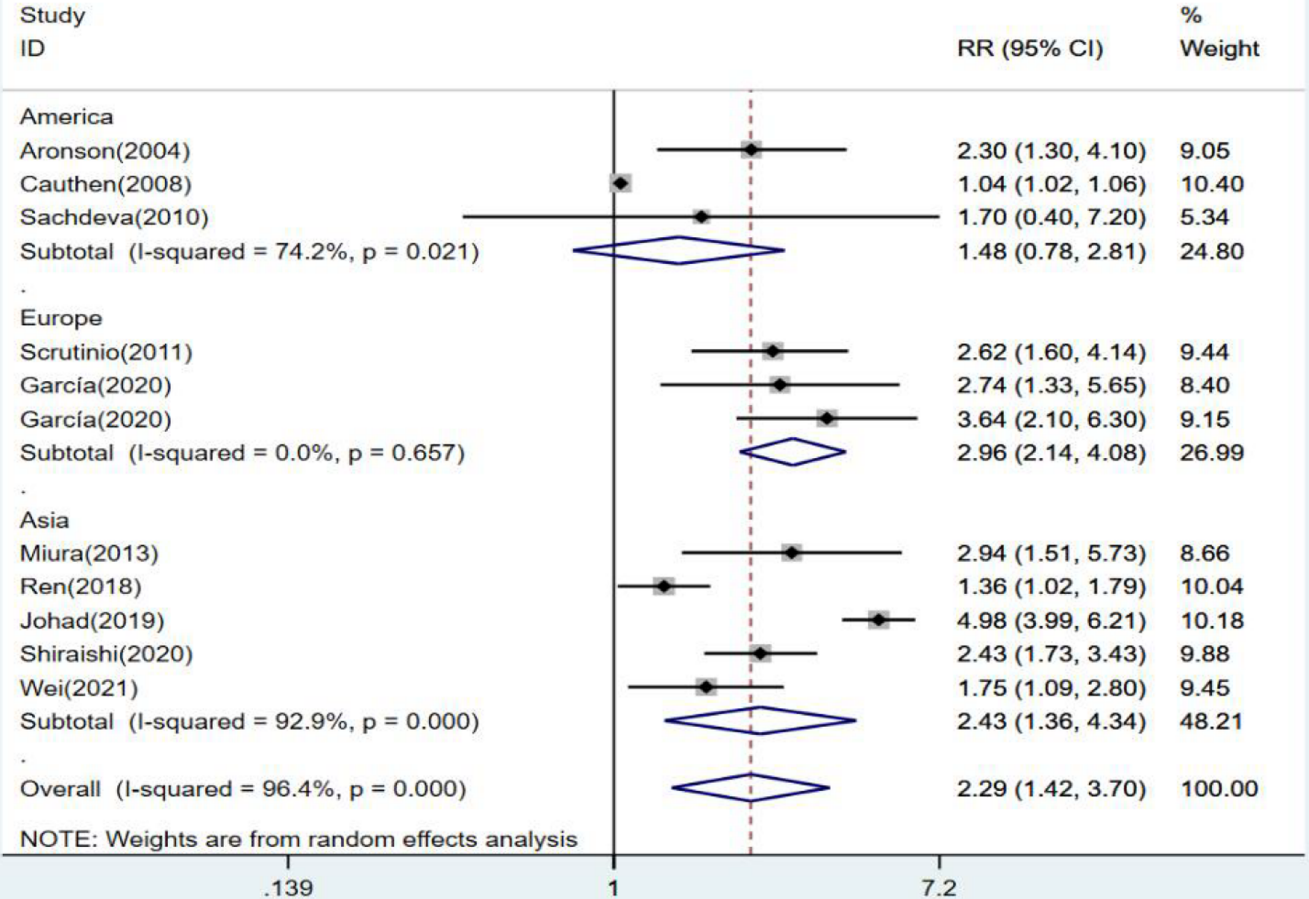
Forest plot of the comparative analysis of blood urea nitrogen levels on the prognosis of patients with heart failure using the random-effects model (among continents); heterogeneity test using *Q*-test and *I*^2^ statistic. CI, confidence interval; RR, relative risk.

When subgroup analysis was performed for each continent, the results revealed no statistical significance in the American group (*RR* = 1.48,95% CI:0.78–2.81, *p* = 0.23). The results were statistically significant in the European group, where the risk of death from heart failure was 2.96 times higher in the high blood urea nitrogen group than in the low group (*RR* = 2.96, 95% CI:2.14–4.08, *p* < 0.001). The risk of death from blood urea nitrogen in the Asian high-level group was 2.43 times higher than that in the low-level group (*RR* = 2.43, 95% CI:1.36–4.34, *p* = 0.003), and the results were statistically significant (Figure 5).

### 3.3. Effect of the blood urea nitrogen as a continuous variable on the prognosis of patients with heart failure

**Figure 6:**
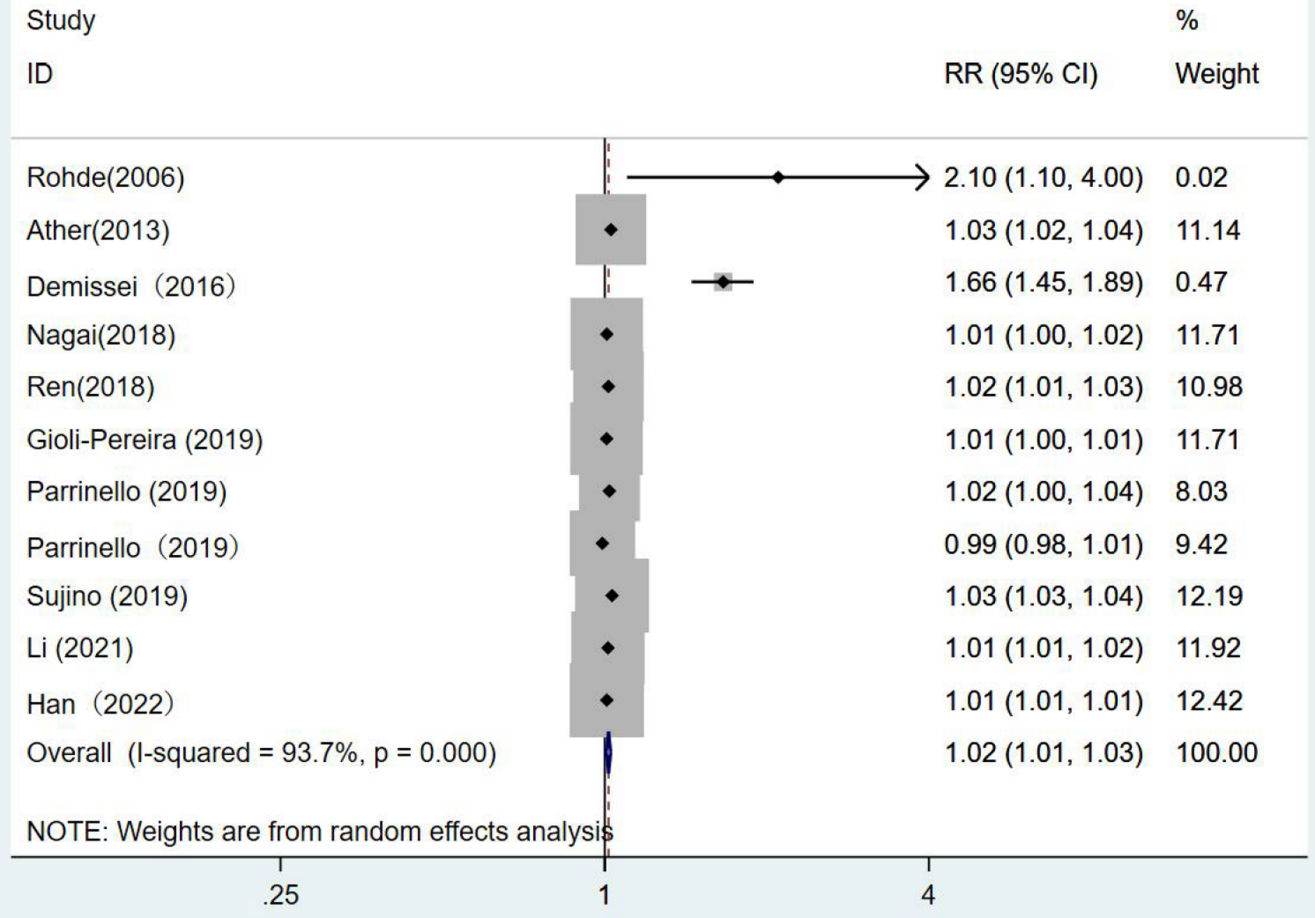
Forest plot of the analysis of blood urea nitrogen levels on the prognosis of patients with heart failure using the random-effects model; heterogeneity test using *Q*-test and *I*^2^ statistic. CI, confidence interval; RR, relative risk.

There were 10 articles using blood urea nitrogen as a continuous variable. The combined *RR* value was 1.02, which was statistically significant (*RR* = 1.02, 95% CI:1.01–1.03, *p* < 0.001). This indicated that the risk of death from heart failure increased by 1.02 times for each unit increase in blood urea nitrogen *I*^2^ = 93.7%. The random-effects model was selected for analysis (Figure 6).

#### 3.3.1. Subgroup analysis

As for the continuous variables, we also explored the sources of heterogeneity by subgroup analysis based on single and multiple factors, different cohort study types, and continents.

**Figure 7:**
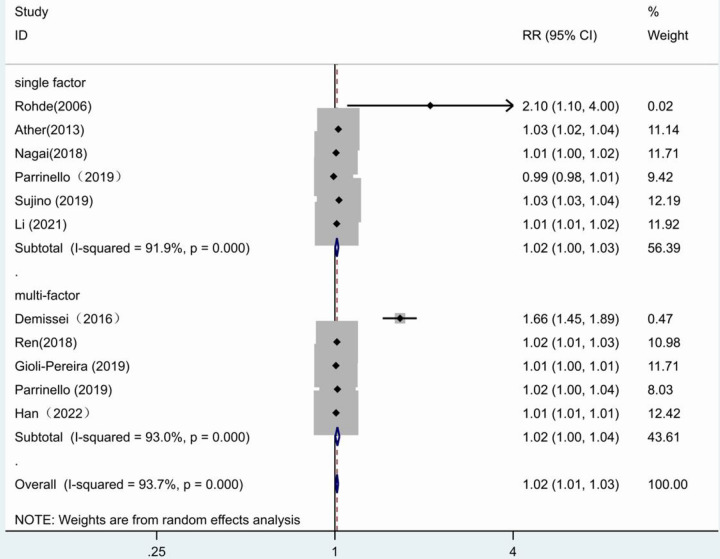
Forest plot of the comparative analysis of blood urea nitrogen levels on the prognosis of patients with heart failure using the random-effects model (single factor vs. multiple factor); heterogeneity test using *Q*-test and *I*^2^ statistic. CI, confidence interval; RR, relative risk.

When univariate and multivariate groups were subdivided, the forest plot results showed no statistical significance in the multivariate group (*RR* = 1.02, 95% CI:1.00–1.04, *p* = 0.017) as well as in the univariate group (*RR* = 1.02, 95% CI:1.00–1.03, *p* = 0.014) (Figure 7).

**Figure 8:**
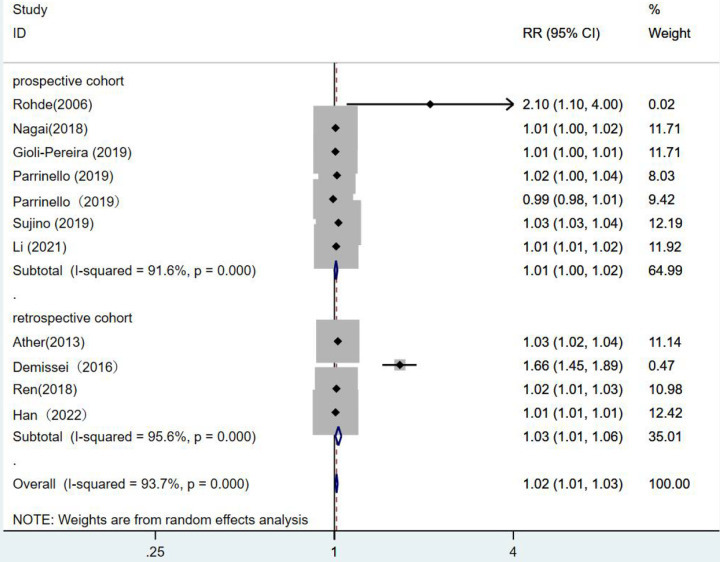
Forest plot of the comparative analysis of blood urea nitrogen levels on the prognosis of patients with heart failure using the random-effects model (prospective vs. retrospective); heterogeneity test using *Q*-test and *I*^2^ statistic. CI, confidence interval; RR, relative risk.

Grouping by study type showed no statistical significance in the prospective cohort studies, (*RR* = 1.01,95% CI:1.00–1.02, *p* = 0.03). In the retrospective cohort studies (*RR* = 1.03,95% CI:1.01–1.06, *p* = 0.009), it was illustrated that a one-unit increase in blood urea nitrogen was associated with a 1.03 fold increase in the risk of death from heart failure (Figure 8).

**Figure 9:**
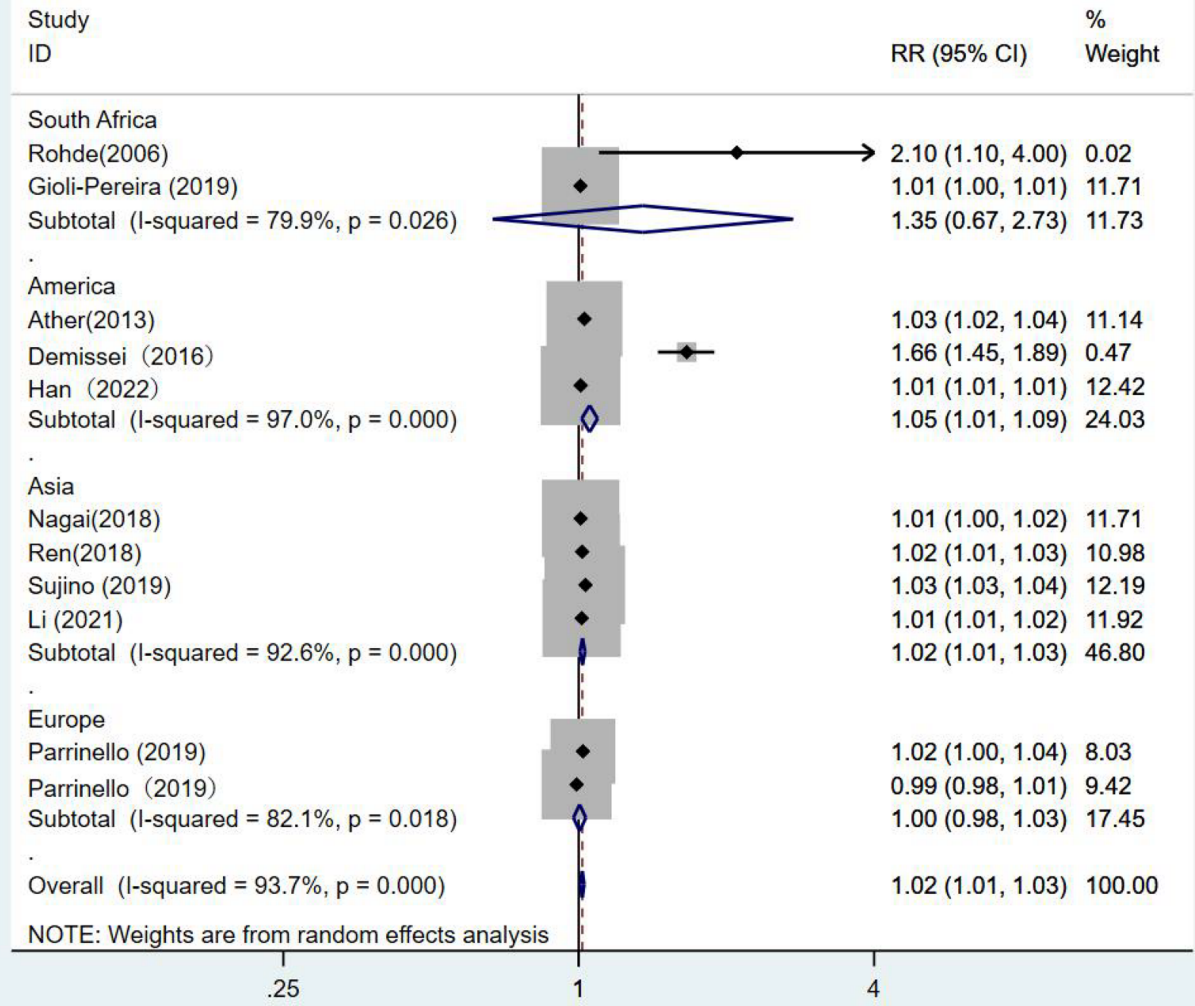
Forest plot of the comparative analysis of blood urea nitrogen levels on the prognosis of patients with heart failure using the random-effects model (among continents); heterogeneity test using *Q*-test and *I*^2^ statistic. CI, confidence interval; RR, relative risk.

When subgroups were divided among continents, the South African group (*RR* = 1.35, 95% CI:0.67–2.73, *p* = 0.402) showed no statistical significance. In the European group (*RR* = 1, 95% CI:0.98–1.03, *p* = 0.779), blood urea nitrogen levels had no statistical significance in predicting death from heart failure. While the results of the American group were statistically significant, as the risk of death from heart failure increased by 1.05 times for each unit increase in blood urea nitrogen (*RR* = 1.05, 95% CI:1.01–1.09, *p* = 0.007). One unit increase in blood urea nitrogen in the Asian group was associated with 1.02 times the risk of death from heart failure. (*RR* = 1.02, 95% CI:1.01–1.03, *p* = 0.003) (Figure 9).

### 3.4. Sensitivity analysis and publication bias

**Figure 10:**
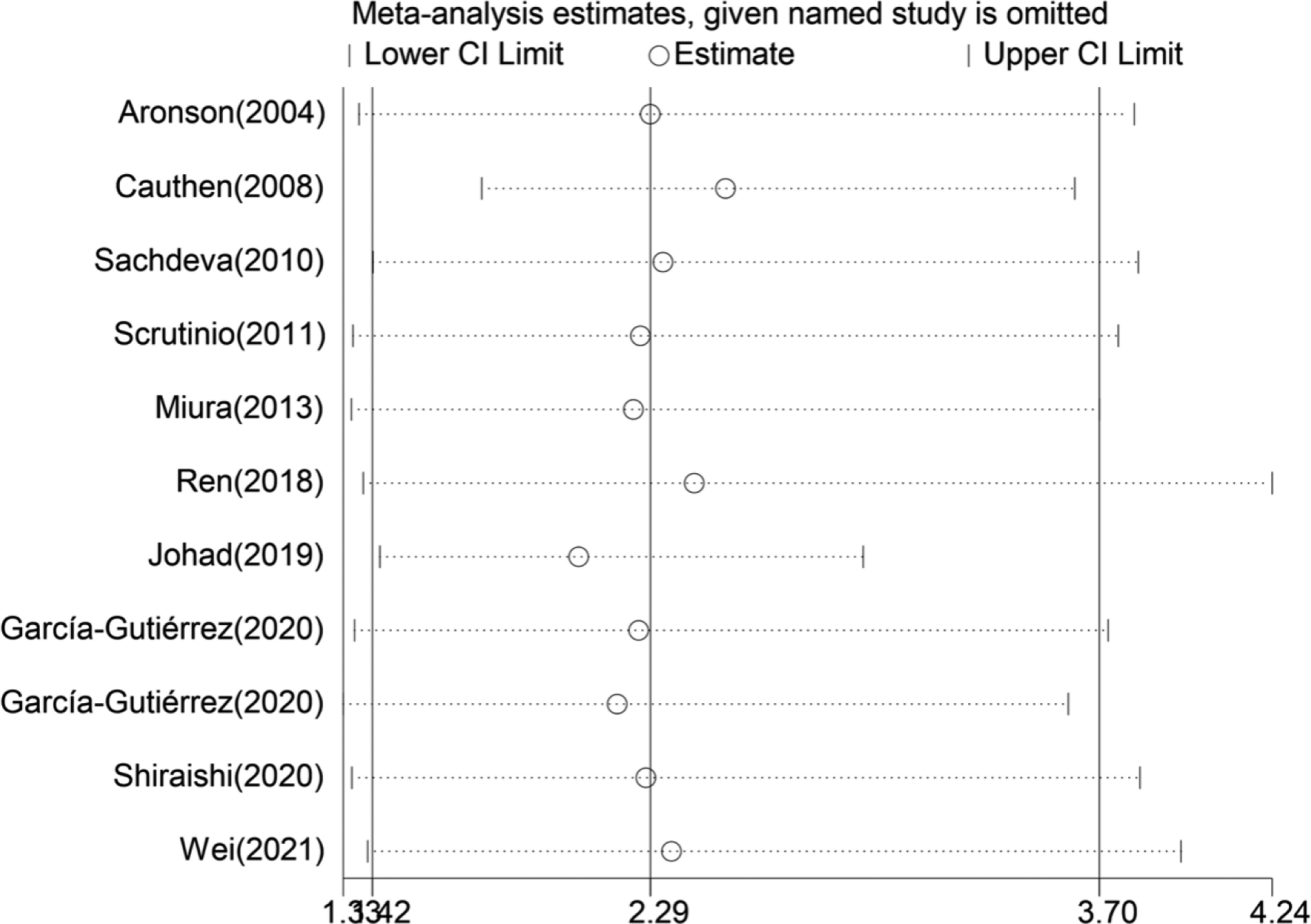
Sensitivity analysis of blood urea nitrogen as a categorical variable on the prognosis of heart failure.

**Figure 11:**
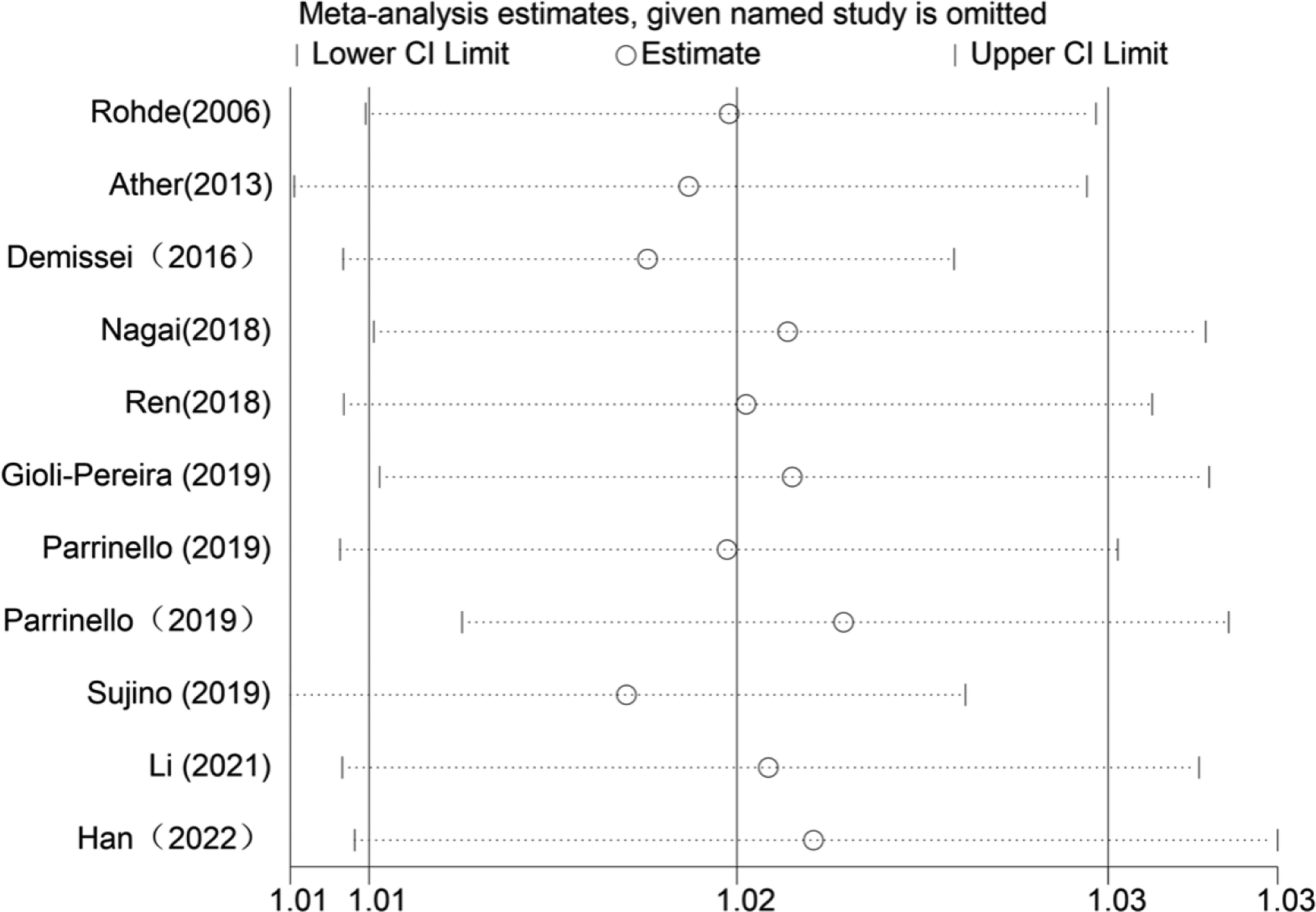
Sensitivity analysis of blood urea nitrogen as a continuous variable on the prognosis of heart failure.

When blood urea nitrogen was analyzed as a categorical and continuous variable, respectively, sensitivity analysis showed that the values of the studies combining the remaining outcomes after removing each group separately remained within the intervals of (95% CI:1.42–3.70) and (95% CI:1.01–1.03), indicating that the stability of blood urea nitrogen levels on all-cause mortality in heart failure patients was not affected by any of the studies (Figures 10, 11).

**Figure 12:**
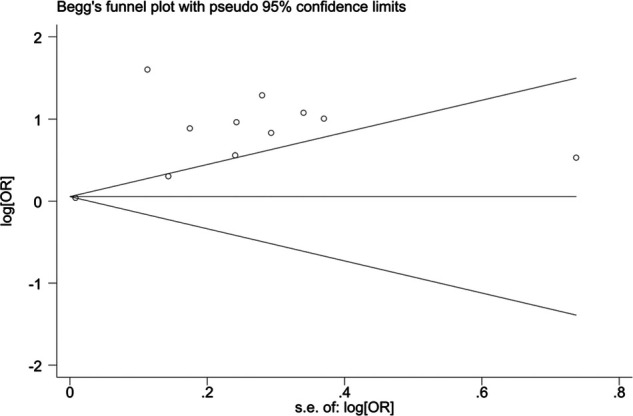
Funnel plot before correction.

**Figure 13:**
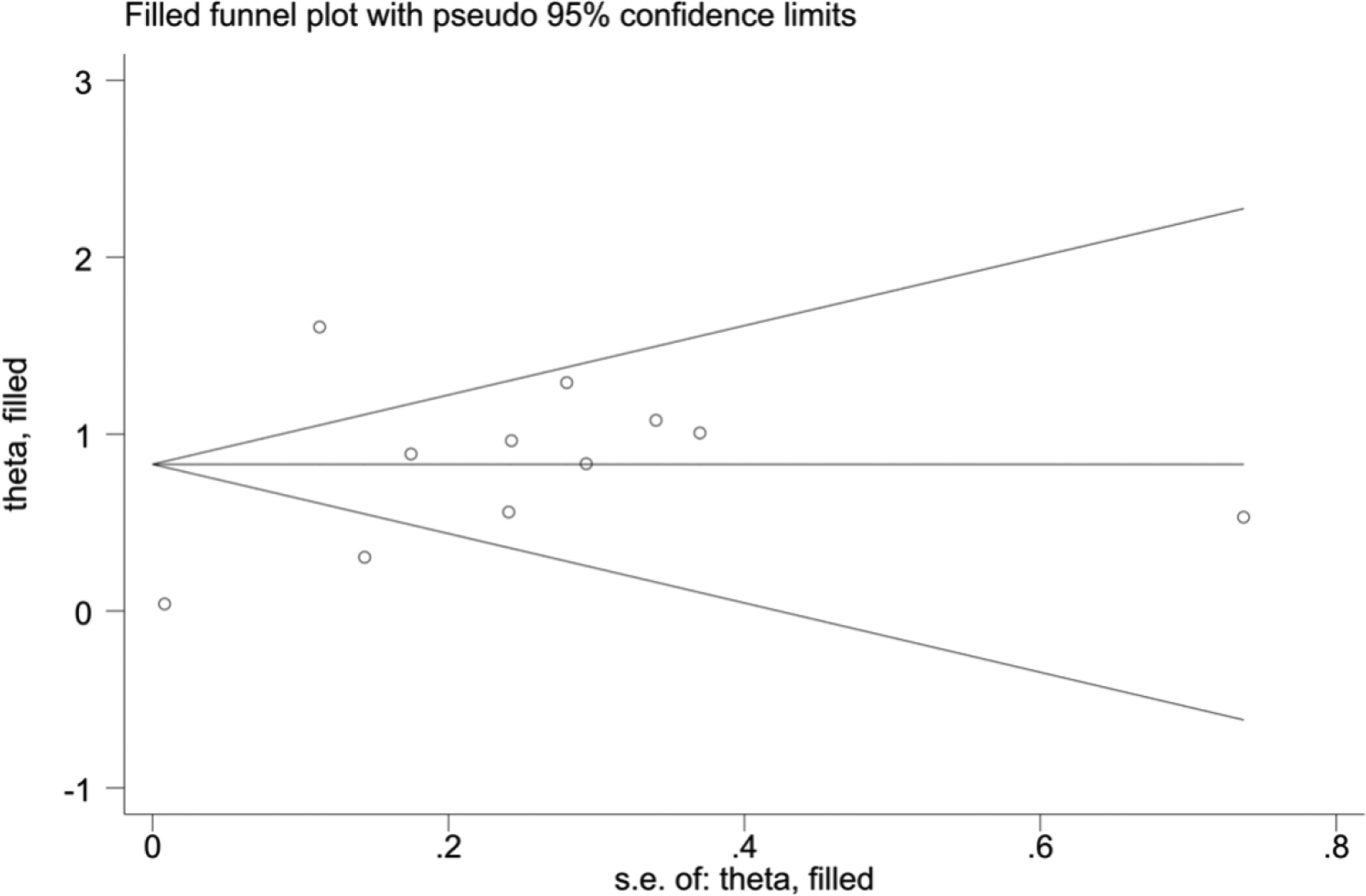
Corrected funnel plot.

**Figure 14:**
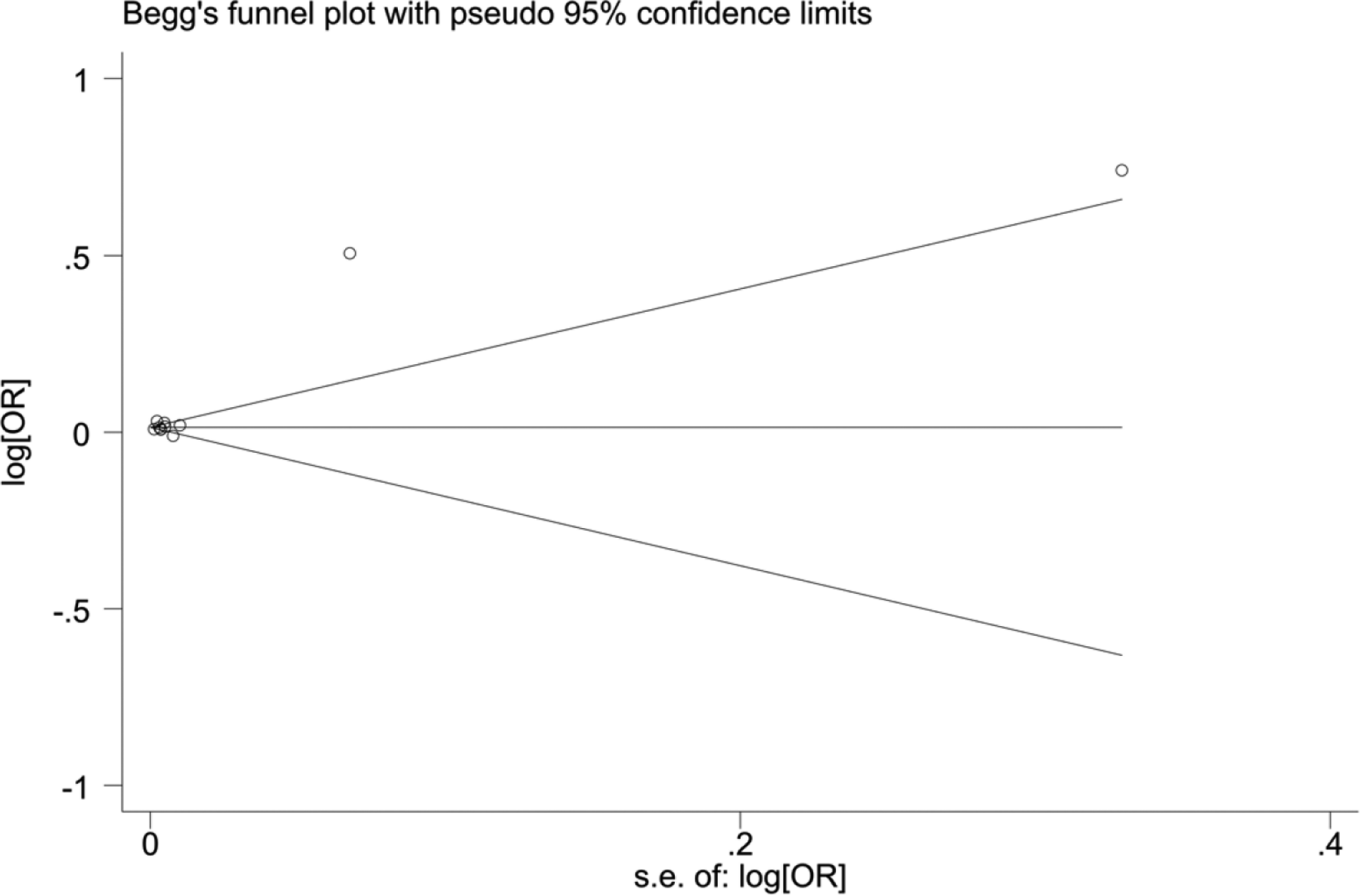
Funnel plot.

When blood urea nitrogen was used as a categorical variable, the funnel plot was asymmetrical, indicating a slight publication bias. Begger's (*p* = 0.64) and Egger's test (*p* = 0.009) suggested a publication bias in Egger's test. To correct for publication bias, we applied the trim and fill method in random effects, and the correction showed a good symmetry of the funnel plot with an *RR* value of 1.057 (95% CI:1.04–1.073). The corrected funnel plot was not significantly different from the initial model, and publication bias did not affect the results (Figures 12, 13). When blood urea nitrogen was used as a continuous variable, Begger's (*p* = 0.35) and Egger's test indicated (*p* = 0.211) that there was no publication bias (Figure 14).

## 4. Discussion

This is the first meta-analysis of the effects of blood urea nitrogen on prognosis of heart failure based on previous research. The pooled results showed that the risk of death from blood urea nitrogen was 2.29 times higher in the high-level group than in the low-level group. And the risk of death from heart failure increased by 1.02 times for every 1 mg/dl increase in blood urea nitrogen. These results indicate a linear relationship between blood urea nitrogen level and death risk of heart failure.

Our findings are consistent with the conclusions of articles by Fonarow et al. (34) and Lombard et al. (35), both suggesting that blood urea nitrogen level is an independent predictor of mortality in patients with heart failure. Additionally, a review conducted by Ouwerkerk et al. has reported that the strongest predictors for mortality in hospitalized patients with heart failure are blood urea nitrogen and sodium (11).

BNP (Brain Natriuretic Peptide) is known to be secreted from ventricular cardiomyocytes. When heart failure occurs, the increased secretion of BNP can inhibit the activation of RASS system, thereby increasing glomerular filtration rate and decreasing the preload of patients with heart failure. A large number of studies have shown that the increase of BNP is associated with the poor prognosis of patients with heart failure, and BNP is the gold standard for the prognosis of early heart failure. In order to explore more biomarkers for heart failure with similar mechanism of action as BNP for clinical use, BUN may be an important indicator for the prognosis of heart failure in the future since the mechanisms of action of BUN and BNP are similar and they had good specificity (36).

Studies have shown that changes in blood urea nitrogen are closely related to the deterioration of renal function in heart failure patients. The prognosis of heart failure can be reflected by monitoring blood urea nitrogen in response to renal perfusion (37). Blood urea nitrogen is a protein metabolite synthesized by the liver and excreted by the kidneys. Hillege et al. suggest that elevated blood urea nitrogen activates the sympathetic nerve and RASS system regulatory mechanism, and participates in the perfusion of renal blood flow. When heart failure occurs, activation of the RASS system promotes water and salt retention, causing proximal tubular reabsorption of urea nitrogen, while sympathetic activation drives distal tubular reabsorption of urea nitrogen, both regulatory mechanisms leading to elevated blood urea nitrogen. Moreover, blood urea nitrogen is also involved in the regulation of antidiuretic hormone. Heart failure stimulates the elevation of antidiuretic hormone, which leads to the concentration of urea nitrogen by the medullary fraction of the kidney, resulting in elevated blood urea nitrogen (38). Therefore, high levels of blood urea nitrogen in hospitalized patients with heart failure have a poor prognosis and require early monitoring and treatment.

In this meta-analysis, due to the large heterogeneity in the forest plot, we conducted analyses exploring the sources of heterogeneity in three subgroups: univariate and multivariate groups, prospective and retrospective cohorts, and different continents. The results showed that the heterogeneity was 89.9% for univariate analysis when blood urea nitrogen was used as a categorical variable, while the size of heterogeneity could not be counted in only one literature for multifactorial analysis. The high heterogeneity in univariate analysis may be due to the fact that indicators such as patient's diet, weight and age influence the prognosis of heart failure patients, resulting in high heterogeneity. Heterogeneity was 98.1% in the retrospective cohort study and 0 in the prospective cohort, suggesting that study type was the source of heterogeneity, which may be owing to the presence of missing patient data and incomplete records in retrospective cohort studies.

Finally, the heterogeneity among continents is 92.9% in Asia, 0 in Europe and 74.2% in America, which may be due to the ethnic variability among individuals, the high level of development in Europe, the inclusion of the same type of heart failure patients, and the low number of deaths from heart failure. In contrast, more patients with heart failure were included in Asia and America with more types of heart failure, leading to high heterogeneity. Heterogeneity in multifactorial analysis was great when blood urea nitrogen was used as a continuous-type variable, which may be related to the different indicators included in each article during the multifactorial analysis. Heterogeneity remained higher in retrospective cohort studies than in prospective cohort studies, with the least heterogeneity in South America when analyzed between continents, which may be a result of South America being in an underdeveloped country with less research on heart failure.

Our study also had limitations. First, the type of heart failure varied among the independent studies included but mainly was acute heart failure, which is a source of clinical variation. Second, multifactorial analysis was not performed in some studies, so whether blood urea nitrogen is an independent predictor of prognostic risk in heart failure remains to be discussed. Finally, patients in these studies were followed up at different time points, which is also a source of variability.

In summary, blood urea nitrogen level is a strong predictor of prognosis in hospitalized patients with heart failure. More research is needed to explore whether lowering blood urea nitrogen could be a therapeutic target for patients with heart failure.

## References

[1] Jackson SL Tong X King RJ Loustalot F Hong Y Ritchey MD. National burden of heart failure events in the United States, 2006 to 2014. Circ Heart Fail. (2018) 11(12):e004873. 10.1161/circheartfailure.117.004873 30562099 PMC6424109

[2] Nazzari H Hawkins NM Ezekowitz J Lauck S Ding L Polderman J The relationship between heart-failure hospitalization and mortality in patients receiving transcatheter aortic valve replacement. Can J Cardiol. (2019) 35(4):413–21. 10.1016/j.cjca.2018.11.016 30853134

[3] Schefold JC Filippatos G Hasenfuss G Anker SD von Haehling S. Heart failure and kidney dysfunction: epidemiology, mechanisms and management. Nat Rev Nephrol. (2016) 12(10):610–23. 10.1038/nrneph.2016.113 27573728

[4] van Veldhuisen DJ Ruilope LM Maisel AS Damman K. Biomarkers of renal injury and function: diagnostic, prognostic and therapeutic implications in heart failure. Eur Heart J. (2016) 37(33):2577–85. 10.1093/eurheartj/ehv588 26543046

[5] Coll E Botey A Alvarez L Poch E Quintó L Saurina A Serum cystatin C as a new marker for noninvasive estimation of glomerular filtration rate and as a marker for early renal impairment. Am J Kidney Dis. (2000) 36(1):29–34. 10.1053/ajkd.2000.8237 10873868

[6] Shenkman HJ Zareba W Bisognano JD. Comparison of prognostic significance of amino-terminal pro-brain natriuretic peptide versus blood urea nitrogen for predicting events in patients hospitalized for heart failure. Am J Cardiol. (2007) 99(8):1143–5. 10.1016/j.amjcard.2006.11.050 17437744

[7] Kazory A. Emergence of blood urea nitrogen as a biomarker of neurohormonal activation in heart failure. Am J Cardiol. (2010) 106(5):694–700. 10.1016/j.amjcard.2010.04.024 20723648

[8] Cleland JG Chiswell K Teerlink JR Stevens S Fiuzat M Givertz MM Predictors of postdischarge outcomes from information acquired shortly after admission for acute heart failure: a report from the placebo-controlled randomized study of the selective A1 adenosine receptor antagonist rolofylline for patients hospitalized with acute decompensated heart failure and volume overload to assess treatment effect on congestion and renal function (protect) study. Circ Heart Fail. (2014) 7(1):76–87. 10.1161/circheartfailure.113.000284 24281134

[9] Klein L Massie BM Leimberger JD O’Connor CM Piña IL Adams KF Jr Admission or changes in renal function during hospitalization for worsening heart failure predict postdischarge survival: results from the outcomes of a prospective trial of intravenous milrinone for exacerbations of chronic heart failure (optime-chf). Circ Heart Fail. (2008) 1(1):25–33. 10.1161/circheartfailure.107.746933 19808267

[10] Sachdeva A Horwich TB Fonarow GC. Comparison of usefulness of each of five predictors of mortality and urgent transplantation in patients with advanced heart failure. Am J Cardiol. (2010) 106(6):830–5. 10.1016/j.amjcard.2010.04.045 20816124

[11] Takaya Y Yoshihara F Yokoyama H Kanzaki H Kitakaze M Goto Y Risk stratification of acute kidney injury using the blood urea nitrogen/creatinine ratio in patients with acute decompensated heart failure. Circ J. (2015) 79(7):1520–5. 10.1253/circj.CJ-14-1360 25854814

[12] Ren X Qu W Zhang L Liu M Gao X Gao Y Role of blood urea nitrogen in predicting the post-discharge prognosis in elderly patients with acute decompensated heart failure. Sci Rep. (2018) 8(1):13507. 10.1038/s41598-018-31059-4 30202087 PMC6131513

[13] Ouwerkerk W Voors AA Zwinderman AH. Factors influencing the predictive power of models for predicting mortality and/or heart failure hospitalization in patients with heart failure. JACC Heart Fail. (2014) 2(5):429–36. 10.1016/j.jchf.2014.04.006 25194294

[14] Rosen R Vandenplas Y Singendonk M. Pediatric gastroesophageal reflux clinical practice guidelines: joint recommendations of the north American society for pediatric gastroenterology, hepatology, and nutrition and the European society for pediatric gastroenterology, hepatology, and nutrition. J Pediatr Gastroenterol Nutr. (2018) 66(3):516–54. 10.1097/MPG.0000000000001889 29470322 PMC5958910

[15] Guyatt GH Oxman AD Kunz R Brozek J Alonso-Coello P Rind D Grade guidelines 6. Rating the quality of evidence–imprecision. J Clin Epidemiol. (2011) 64(12):1283–93. 10.1016/j.jclinepi.2011.01.012 21839614

[16] Aronson D Mittleman MA Burger AJ. Elevated blood urea nitrogen level as a predictor of mortality in patients admitted for decompensated heart failure. Am J Med. (2004) 116(7):466–73. 10.1016/j.amjmed.2003.11.014 15047036

[17] Cauthen CA Lipinski MJ Abbate A Appleton D Nusca A Varma A Relation of blood urea nitrogen to long-term mortality in patients with heart failure. Am J Cardiol. (2008) 101(11):1643–7. 10.1016/j.amjcard.2008.01.047 18489944

[18] Scrutinio D Passantino A Catanzaro R Guida P. Clinical utility of different estimates of renal function for predicting mortality in chronic heart failure. Int J Cardiol. (2012) 157(1):24–30. 10.1016/j.ijcard.2010.10.131 21115207

[19] Miura M Sakata Y Nochioka K Takahashi J Takada T Miyata S Prognostic impact of blood urea nitrogen changes during hospitalization in patients with acute heart failure syndrome. Circ J. (2013) 77(5):1221–8. 10.1253/circj.cj-12-1390 23392088

[20] Khoury J Bahouth F Stabholz Y Elias A Mashiach T Aronson D Blood urea nitrogen variation upon admission and at discharge in patients with heart failure. ESC Heart Fail. (2019) 6(4):809–16. 10.1002/ehf2.12471 31199082 PMC6676277

[21] Khoury J Bahouth F Stabholz Y Elias A Mashiach T Aronson D Blood urea nitrogen variation upon admission and at discharge in patients with heart failure. ESC Heart Fail. (2019) 6(4):809–16. 10.1002/ehf2.12471 31199082 PMC6676277

[22] García-Gutiérrez S Antón-Ladislao A Quiros R Lara A Rilo I Morillas M Short-term mortality risk score for de novo acute heart failure (essic-fehf). Eur J Intern Med. (2020) 77:52–8. 10.1016/j.ejim.2020.02.012 32145979

[23] Shiraishi Y Kohsaka S Abe T Nagai T Goda A Nishihata Y Derivation and validation of clinical prediction models for rapid risk stratification for time-sensitive management for acute heart failure. J Clin Med. (2020) 9(11):3394. 10.3390/jcm9113394 33113911 PMC7690673

[24] Wei X Min Y Yu J Wang Q Wang H Li S The value of admission serological indicators for predicting 28-day mortality in intensive care patients with acute heart failure: construction and validation of a nomogram. Front Cardiovasc Med. (2021) 8:741351. 10.3389/fcvm.2021.741351 34926602 PMC8678052

[25] Rohde LE Goldraich L Polanczyk CA Borges AP Biolo A Rabelo E A simple clinically based predictive rule for heart failure in-hospital mortality. J Card Fail. (2006) 12(8):587–93. 10.1016/j.cardfail.2006.06.475 17045176

[26] Ather S Bavishi C McCauley MD Dhaliwal A Deswal A Johnson S Worsening renal function is not associated with response to treatment in acute heart failure. Int J Cardiol. (2013) 167(5):1912–7. 10.1016/j.ijcard.2012.05.004 22633437 PMC4682884

[27] Demissei BG Valente MA Cleland JG O’Connor CM Metra M Ponikowski P Optimizing clinical use of biomarkers in high-risk acute heart failure patients. Eur J Heart Fail. (2016) 18(3):269–80. 10.1002/ejhf.443 26634889

[28] Nagai T Yoshikawa T Saito Y Takeishi Y Yamamoto K Ogawa H Clinical characteristics, management, and outcomes of Japanese patients hospitalized for heart failure with preserved ejection fraction- a report from the Japanese heart failure syndrome with preserved ejection fraction (jasper) registry. Circ J. (2018) 82(6):1534–45. 10.1253/circj.CJ-18-0073 29576598

[29] Gioli-Pereira L Marcondes-Braga FG Bernardez-Pereira S Bacal F Fernandes F Mansur AJ Predictors of one-year outcomes in chronic heart failure: the portrait of a middle income country. BMC Cardiovasc Disord. (2019) 19(1):251. 10.1186/s12872-019-1226-9 31706288 PMC6842241

[30] Parrinello G Torres D Buscemi S Di Chiara T Cuttitta F Cardillo M Right ventricular diameter predicts all-cause mortality in heart failure with preserved ejection fraction. Intern Emerg Med. (2019) 14(7):1091–100. 10.1007/s11739-019-02071-x 30895427

[31] Sujino Y Nakano S Tanno J Shiraishi Y Goda A Mizuno A Clinical implications of the blood urea nitrogen/creatinine ratio in heart failure and their association with haemoconcentration. ESC Heart Fail. (2019) 6(6):1274–82. 10.1002/ehf2.12531 31814319 PMC6989280

[32] Li Y Sun XL Qiu H Qin J Li CS Yu XZ Long-term outcomes and independent predictors of mortality in patients presenting to emergency departments with acute heart failure in Beijing: a multicenter cohort study with a 5-year follow-up. Chin Med J. (2021) 134(15):1803–11. 10.1097/cm9.0000000000001617 34224408 PMC8367075

[33] Han D Xu F Zhang L Yang R Zheng S Huang T Early prediction of in-hospital mortality in patients with congestive heart failure in intensive care unit: a retrospective observational cohort study. BMJ. (2022) 12(7):e059761. 10.1136/bmjopen-2021-059761

[34] Fonarow GC Adams KF Jr Abraham WT Yancy CW Boscardin WJ. Risk stratification for in-hospital mortality in acutely decompensated heart failure: classification and regression tree analysis. JAMA. (2005) 293:572–80. 10.1001/jama.293.5.572 15687312

[35] Lombardi C Carubelli V Rovetta R Castrini AI Vizzardi E Bondei I Prognostic value of serial measurements of blood urea nitrogen in ambulatory patients with chronic heart failure. Panminerva Med. (2016) 58(1):8–15. 26154625

[36] Castiglione V Aimo A Vergaro G Saccaro L Passino C Emdin M. Biomarkers for the diagnosis and management of heart failure. Heart Fail Rev. (2022) 27(2):625–43. 10.1007/s10741-021-10105-w 33852110 PMC8898236

[37] Ruocco G Pellegrini M De Gori C Franci B Nuti R Palazzuoli A. The prognostic combined role of B-type natriuretic peptide, blood urea nitrogen and congestion signs persistence in patients with acute heart failure. J Cardiovasc Med. (2016) 17(11):818–27. 10.2459/jcm.0000000000000350 26702597

[38] Hillege HL Girbes AR de Kam PJ Boomsma F de Zeeuw D Charlesworth A Renal function, neurohormonal activation, and survival in patients with chronic heart failure. Circulation. (2000) 102(2):203–10. 10.1161/01.cir.102.2.203 10889132

